# Researcher and study participants’ perspectives of consent in clinical studies in four referral hospitals in Vietnam

**DOI:** 10.1186/s12910-020-0445-z

**Published:** 2020-01-10

**Authors:** Jennifer Ilo Van Nuil, Thi Thanh Thuy Nguyen, Thanh Nhan Le Nguyen, Van Vinh Chau Nguyen, Mary Chambers, Thi Dieu Ngan Ta, Laura Merson, Thi Phuong Dung Nguyen, Minh Tu Van Hoang, Michael Parker, Susan Bull, Evelyne Kestelyn

**Affiliations:** 1 Oxford University Clinical Research Unit, Hospital for Tropical Disease, 764 Vo Van Kiet Street, District 5, Ho Chi Minh City, Vietnam; 2 Nuffield Department of Medicine, Centre for Tropical Medicine and Global Health, University of Oxford, Oxford, UK; 3 Children’s Hospital 1, Ho Chi Minh City, Vietnam; 4 Hospital for Tropical Diseases, Ho Chi Minh City, Vietnam; 5 National Hospital for Tropical Diseases, Hanoi, Vietnam; 6 Infectious Diseases Data Observatory, Oxford, UK; 7 Children Hospital 2, Ho Chi Minh City, Vietnam; 8 Ethox Centre, University of Oxford, Oxford, UK

**Keywords:** Consent, Research ethics, Clinical research, *nghiên cứu*, Trust, Vietnam

## Abstract

**Background:**

Within the research community, it is generally accepted that consent processes for research should be culturally appropriate and tailored to the context, yet researchers continue to grapple with what valid consent means within specific stakeholder groups. In this study, we explored the consent practices and attitudes regarding essential information required for the consent process within hospital-based trial communities from four referral hospitals in Vietnam.

**Methods:**

We collected surveys from and conducted semi-structured interviews with study physicians, study nurses, ethics committee members, and study participants and family members regarding their experiences of participating in research, their perspectives toward research, and their views about various elements of the consent process.

**Results:**

In our findings, we describe three interrelated themes related to the consent process: (1) words and regulation; (2) reimbursement, suspicions, and joining; and (3) responsibilities. In general, stakeholders had highly varied perspectives of *nghiên cứu* (Eng.: research) and researchers used varying levels of detail regarding all aspects of the study in the consent process to build trust with and/or promote potential research participants’ choices about taking part in research. Findings additionally highlight how researchers felt that offering financial reimbursements in a hospital setting, where payment for services was routine, would be unfamiliar to participants and could raise suspicions about the research. Participants, however, focused their discussions on reimbursement or alternative reasons for joining the study, such as health related benefits or altruism. Finally, participants often relied on their physician to help them decide about joining a study or not.

**Conclusion:**

Further research is needed to understand how researchers and participants make sense of and practice consent, and how that impacts participants’ decision-making about research participation. To promote valid consent within this context, it is important to engage with hospital-based trial communities as a whole. The data from this study will inform future research on consent, guide the revisions of consent related policies within our research sites and point to several larger issues surrounding researcher-participant expectations, communication, and trust.

## Background

Although the research community generally accepts that the consent process for clinical research should be culturally appropriate and tailored to the context, researchers continue to grapple with what valid consent means for research within specific communities and stakeholder groups [1]. Clinical studies that take place within a hospital setting have a unique set of complexities as potential participants often have to make choices about research participation in the context of severe illnesses. The Council for International Organizations of Medical Sciences (CIOMS) defines a valid consent process as providing “potential research participants with the information and the opportunity to give their free and informed consent to participate in research” emphasizing processes that protect free choice and respect individual autonomy [2]. In practice, the amount of information that is considered sufficient is not easily defined, understanding varies amongst and within communities, and choices (individual and otherwise) are influenced by a variety of factors that may not be obvious.

Developing a consent process with the right amount of information for sufficient understanding continues to be a challenge for researchers, especially with complex study designs. Several methods have been designed to adapt the consent process to specific contexts using, for example, an enhanced consent process that reduces the amount of information presented while maintaining international regulations [3] or rapid ethical assessments prior to development of specific consent processes [4, 5]. In a recent review, Gillies (2018) synthesized papers that focused on patient reported measures of consent in clinical trials and found that the majority of measures focused on understanding, not the myriad factors that influence participation, such as decision-making mechanisms [6].

The multiple structural factors that shape individuals’ lives also impact the decisions that people make and at times, it becomes difficult to assess the voluntariness of such decisions [7]. In some contexts, participants decide to join a study prior to the consent process, based on informal information circulating in the community about the personal and health benefits of joining the study [8–10]. In contexts where research is the one of few routes to access (better) healthcare, the “anticipated therapeutic benefit” may overshadow study risks or enhance perceived study benefits, yet not offering enough benefits can result in exploitation [11]. Further, complex gender and power dynamics can impact whether and how individuals make choices about participation in studies [12–14]. There is a growing body of literature surrounding trust and how it can shape the consent process and acceptance of joining research studies [15, 16]. Communication and rumors about research, illnesses, and the individual studies circulate within and between communities and the broader public moving the communication outside the realm of the research sites. Researchers have noted that rumors about blood and other medical procedures can reduce trial recruitment and retention [14, 17, 18]. Formal and informal information about studies both shape perceptions of research and decision-making processes for joining a study [19]. These findings highlight the importance of acknowledging the context beyond the consent session itself when trying to understand what consent means to potential participants, research staff and ethics committee members.

Based on experiences within a clinical trial site in Vietnam, we noted potential gaps in how hospital-based research was understood by study participants, how consent was practiced and received at study sites, and what consent meant to all stakeholders, despite the increasing amount of clinical research occurring in the context. In this study, we explored the range of perspectives surrounding consent within hospital-based trial communities, as well as the attitudes regarding the essential elements of consent.

## Methods

### Study setting

The study took place within four partner hospitals working with Oxford University Clinical Research Unit (OUCRU) in Vietnam. OUCRU formed one of the earliest collaborations with the Hospital for Tropical Diseases in Ho Chi Minh City in 1991 and now engages in numerous clinical trials and studies with several institutions across Vietnam. The most recent Vietnamese Ministry of Health National Ethics Guidelines for Biomedical Research, released in 2013, include content requirements for clinical trial consent forms and basic principles for obtaining consent. To supplement the Ministry of Health guidelines, OUCRU developed a standard consent process.

## Study design and procedures

We used a cross sectional approach and collected data using surveys and semi-structured interviews. The semi-structured interviews covered three topics including: (i) interviewees’ experiences related to their role in the research process, (ii) attitudes regarding inclusion of the elements of consent as described in the International Conference of Harmonization Good Clinical Practice (ICH-GCP) guidelines, section E6 4.8 [20], and (iii) challenges and proposed solutions regarding the content of the consent process. In the survey, the elements from ICH-GCP section E6 were listed and the participants were asked to rank the importance of each element for inclusion in the consent form from 1 to 3, with 1 indicating essential information, 2 indicating a neutral view, and 3 indicating non-essential information.

A mixture of purposive and convenience sampling was used in this study in order to obtain a range of experiences related to consent. We recruited a variety of stakeholders including hospital EC members, physicians, nurses (referred to as ‘researchers’ throughout) and study participants and family members (referred to as ‘participants’ throughout). To ensure a baseline level of experience with research, we selected researchers who had worked on at least two clinical studies in the hospital context. For participants, we included adults who had participated or were actively participating in a study, and parents or relatives of children who had participated in clinical research within the past 6 months or were actively participating in clinical research. Participants were drawn from two studies that were being conducted within the four hospitals. Both studies focused on severe dengue and recruited from the outpatient departments of the hospitals, and one study also recruited from the inpatient department. One study focused solely on children ages 1–15 and the other study included both children and adults. We recruited researchers from the same hospitals, and related research and medical institutions, however, the researchers worked on a broader range of research and not necessarily the studies from which participants were recruited.

NTTT interviewed all stakeholders in Vietnamese using a semi-structured interview guide. The interviews took place in settings within the hospital that would protect the stakeholders’ privacy as much as possible, or a place where the stakeholder felt most comfortable. All interviews were audio-recorded, with stakeholders’ consent. Prior to the interview with participants, we provided the information sheet for the dengue studies and it was available to them during the interview for reference. We administered the same survey and asked the same main interview questions to all stakeholders, and probed specific topics when necessary. We conducted two pilot interviews prior to beginning the study to test the questions and refine the interview guide.

The survey results were documented and presented as frequencies and stratified by group (participants or researchers). NTTT transcribed the interviews verbatim from the audio recordings and a translator translated it from Vietnamese to English. Data were de-identified by removing specific names or places that could potentially identify participants. After verification of translations, data were imported into NVivo 12, software for organization and coding. After reviewing the interview summaries and reading the transcripts, we created an initial codebook based on a set of core themes from the research questions. We then coded the interview transcripts with these codes. Next, we grouped the topic responses from each area into smaller codes using an inductive coding approach. We used thematic analysis to identify larger themes across all interviews. In this paper, we discuss three main themes, (1) words and regulation; (2) reimbursement, suspicions, and joining; and (3) responsibilities. The study tools are included in Additional file 1 and interview transcripts are available upon request, following the standard OUCRU data sharing policy.

The ethics committees of the Hospital for Tropical Diseases, Children’s Hospital 1, and Children’s Hospital 2 all located in Ho Chi Minh City, Vietnam and the National Hospital for Tropical Diseases in Hanoi, Vietnam all reviewed and approved this protocol. All potential stakeholders were provided with explanations of the purpose of the study, procedures, risks, benefits, and alternatives to study participation. Written consent was obtained from all stakeholders prior to the survey and interview, as required by the local research ethics committees.

## Results

In total, 41 individuals participated in the study from July 2013 to December 2014. We interviewed four past or current dengue study participants and 10 family members or relatives of child participants. Their ages ranged from 21 to 57 (median age 33) and 57.1% (8/14) were female (see Table 1). All interviews took place within the hospital with the exception of one interview, which took place in the participant’s home. The primary occupation of participants varied widely, and included housewives, business owners, nurses, teachers, and tailors. Interviews were conducted between 2 weeks and 6 months of the most recent study visit. Data collection with researchers comprised interviews with 13 physicians who were not members of the hospital ethics committee, 11 physicians who also served as hospital ethics committee members, two hospital ethics committee members who were not physicians, and one nurse. Forty-four percent (12/27) were female and their ages ranged from 30 to 61 (median age 43). The majority (88.8%) of the researchers worked within a national hospital setting, either in Hanoi or Ho Chi Minh City (see Table 2). Overall, five people declined to participate in the study, all stating that the interview timing was inconvenient.

**Table 1:** Characteristics of study participant stakeholders

	*N* = 14
Age, median years, (IQR)	33 (30, 40)
Gender, number (%)	
Women	8 (57.1)
Men	6 (42.9)
Study Role, number (%)	
Participant	4 (28.6)
Patient representative	10 (71.4)
Institution from where recruited, number (%)	
Infectious Disease Hospitals	5 (35.7)
Children’s Hospitals	6 (42.9)
Unknown	3 (21.4)
Disease under study, number (%)	
Dengue	14 (100.0)

**Table 2:** Characteristics of research stakeholders

	*N* = 27
Age, median years (IQR)	43 (32, 55)
Gender, number (%)	
Women	12 (44.4)
Men	15 (55.6)
Role, number (%)	
Physician	13 (48.1)
Physician & EC member	11 (40.7)
EC member	2 (7.4)
Nurse	1 (3.7)
Institution, number (%)	
Infectious Disease Hospital	12 (44.4)
Children’s Hospital	12 (44.4)
Medical Center	1 (3.7)
Research Institute	1 (3.7)
Ministry of Health	1 (3.7)

In the following text, we describe three key themes surrounding the perspectives about, and experiences with, the consent process identified during analysis including: (1) words and regulation; (2) reimbursement, suspicions, and joining; and (3) responsibilities. These three themes encompass varying levels of the elements of valid consent including sufficient information, knowledge and understanding, and the protection of free choice. Within each theme, we explore relevant quantitative findings from the survey; the complete survey results are set out in Table 3.

**Table 3:** Ranking of essential elements of consent

	Research Stakeholders *N* = 27	Study Participants (*N* = 14)	Total (*N* = 41)
Inclusion of the word research			
Essential	19 (70.4)	10 (71.4)	29 (70.7)
Neutral	6 (22.2)	2 (14.3)	8 (19.5)
Non-essential	2 (7.4)	2 (14.3)	4 (9.8)
Study purpose			
Essential	24 (88.9)	13 (92.9)	37 (90.2)
Neutral	3 (11.1)	1 (7.1)	4 (9.8)
Non-essential	0 (0.0)	0 (0.0)	0 (0.0)
Use of randomization			
Missing	0	2	2
Essential	8 (33.3)	5 (41.7)	13 (33.3)
Neutral	8 (29.6)	3 (25.0)	11 (28.2)
Non-essential	11 (37.0)	4 (33.3)	15 (38.5)
Study procedures			
Essential	19 (70.4)	10 (71.4)	29 (70.7)
Neutral	7 (25.9)	3 (21.4)	10 (24.4)
Non-essential	1 (3.7)	1 (7.1)	2 (4.9)
Participant responsibilities			
Essential	18 (66.7)	12 (85.7)	30 (73.2)
Neutral	8 (29.6)	0 (0.0)	8 (19.5)
Non-essential	1 (3.7)	2 (14.3)	3 (7.3)
Study risks			
Essential	18 (66.7)	7 (50.0)	25 (61.0)
Neutral	8 (29.6)	5 (35.7)	13 (31.7)
Non-essential	1 (3.7)	2 (14.3)	3 (7.3)
Study benefits			
Missing	0	1	1
Essential	19 (70.4)	10 (76.9)	29 (72.5)
Neutral	6 (22.2)	2 (15.4)	8 (20.0)
Non-essential	2 (7.4)	1 (7.7)	3 (7.5)
Alternative treatments			
Missing	0	1	1
Essential	13 (48.1)	10 (76.9)	23 (57.5)
Neutral	8 (29.6)	3 (23.1)	11 (27.5)
Non-essential	6 (22.2)	0 (0.0)	6 (15.0)
Insurance			
Missing	1	0	1
Essential	10 (38.5)	10 (71.4)	20 (50.0)
Neutral	10 (38.5)	2 (14.3)	12 (30.0)
Non-essential	6 (23.1)	2 (14.3)	8 (20.0)
Payments			
Essential	16 (59.3)	8 (57.1)	24 (58.5)
Neutral	8 (29.6)	2 (14.3)	10 (24.4)
Non-essential	3 (11.1)	4 (28.6)	7 (17.1)
Study costs			
Missing	0	1	1
Essential	16 (59.3)	11 (84.6)	27 (67.5)
Neutral	7 (25.9)	1 (7.7)	8 (20.0)
Non-essential	4 (14.8)	1 (7.7)	5 (12.5)
Voluntary nature of study			
Missing	0	1	1
Essential	23 (85.2)	10 (76.9)	33 (82.5)
Neutral	4 (14.8)	1 (7.7)	5 (12.5)
Non-essential	0 (0.0)	2 (15.4)	2 (5.0)
Data access by monitors			
Missing	0	1	1
Essential	5 (18.5)	3 (23.1)	8 (20.0)
Neutral	2 (7.4)	2 (15.4)	4 (10.0)
Non-essential	20 (74.1)	8 (61.5)	28 (70.0)
Confidentiality			
Missing	0	1	1
Essential	15 (55.6)	6 (46.2)	21 (52.5)
Neutral	9 (33.3)	2 (15.4)	11 (27.5)
Non-essential	3 (11.1)	5 (38.5)	8 (20.0)
Inform when changes to protocol			
Missing	0	1	1
Essential	6 (22.2)	11 (84.6)	17 (42.5)
Neutral	10 (37.0)	2 (15.4)	12 (30.0)
Non-essential	11 (40.7)	0 (0.0)	11 (27.5)
Contact information of investigators			
Missing	0	1	1
Essential	19 (63.0)	11 (84.6)	30 (75.0)
Neutral	6 (22.2)	0 (0.0)	6 (15.0)
Non-essential	2 (14.8)	2 (15.4)	4 (10.0)
Study termination			
Missing	0	2	2
Essential	6 (22.2)	3 (25.0)	9 (23.1)
Neutral	6 (22.2)	1 (8.3)	7 (17.9)
Non-essential	15 (55.6)	8 (66.7)	23 (59.0)
Study duration			
Essential	16 (59.3)	9 (64.3)	25 (61.0)
Neutral	3 (11.1)	2 (14.3)	5 (12.2)
Non-essential	8 (29.6)	3 (21.4)	11 (26.8)

### Words and regulation

We asked all stakeholders about their attitudes regarding the meaning and implications of using the word *nghiên cứu* (Eng.: research or study), a word that is mandated by ICH-GCP to be included in the consent forms and that should be discussed explicitly during the consent process [20]. From the survey data, 71.4% (10/14) of participants and 70.4% (19/27) of researchers stated that it was essential to include the exact word *nghiên cứu* in the consent forms; only two researchers and two participants thought that it was non-essential information. Stakeholders had highly varied perspectives of the meaning of *nghiên cứu,* which led to conversations about the level of detail regarding research that should be discussed in the consent process.

Overall, stakeholders’ discussions about the meaning of *nghiên cứu* were rather negative. During interviews, 17 researchers and six participants mentioned negative connotations regarding *nghiên cứu*, including “guinea pigs” or “lab rats”, “testing”, “invasiveness”, “being used”, and “being experimented on.” They also discussed feelings of fear and anxiety related with *nghiên cứu* because it sounded “scary” and “heavy” and if used in consent processes, would need careful explanations. Researchers relayed their opinions and experiences regarding how they thought potential participants viewed research, while participants spoke of their own and other community members’ perspectives.

*Vietnamese tend to avoid* nghiên cứu *since they consider being in experiments the equivalent of serving as a guinea pig. We should avoid using words such as* nghiên cứu or *“experiment”, and so on. [Physician 37]*

*If they don’t understand deeply, if you say* nghiên cứu*, they will be scared.* Nghiên cứu *sounds so heavy. Sometimes, for the people [with lower education], the term* nghiên cứu *will make them scared. [Patient Representative 06]*

This same participant (06) consented for her child to participate in dengue research although it was not clear to her why a blood test would be considered to be research, rather than a diagnostic measure: “[Research] makes me scared… so don’t use the *nghiên cứu* word for testing blood only.” The delineation of clinical care and *nghiên cứu* was not clear for this patient representative.

There were, however, two participants who had more positive views on *nghiên cứu,* linking it to trust in research and science.

*Because when people hear* nghiên cứu *is related to science, they will feel more trust [than they do with other disciplines]… because science often has factuality. If some organization supports the programme, it is much better and safer than if some individuals do [the research]. [Study Participant 32]*

In addition to discussing the meaning of *nghiên cứu,* researchers spoke about how to discuss *nghiên cứu* during consent sessions, often in the context of being honest or regarding the level of detail about research that should be provided for consent to be considered appropriately informed. For example, a study doctor stressed how researchers must be honest with potential participants both as a moral obligation and to obtain more honest responses from participants.

*I think we should be honest with patients. I mean we should tell them about our research because it requires cooperation to get better results. Obviously we should tell the patients our work is* nghiên cứu*. [Physician & EC Member 35]*

For this researcher, being clear about the nature of the study enhanced the cooperation between the participants and the researchers and was advantageous for the overall quality of data. However, throughout the remainder of the interview, she stated that although *nghiên cứu* should be discussed to some extent, researchers should balance the amount of information discussed during consent for two main reasons: confusion and fear. If the consent process left the potential participants confused and scared, fewer potential participants were likely to consent to research. Other research stakeholders echoed similar attitudes about the amount of information that should be communicated in the consent process. They felt that the researchers should provide just enough information so that the participants feel “comfortable” and “safe” but not too much so that they become scared of the research. The survey findings similarly show that ‘study purpose’ was considered essential by 88.9% (24/27) of researchers, ‘study procedures’ were considered essential by 70.4% (19/27), while ‘use of randomization’ or ‘protocol changes’ were considered essential by only 33.3% (8/27) and 22.2% (6/27) respectively. It was difficult to determine from the interview data if providing limited information was a way to provide appropriate levels of information to a population with limited understanding or if it was a strategy to obscure elements of the study that might scare participants, or a combination of both.

There were two researchers who stated that the amount of information provided during consent did not matter at all, as long as the participants had trust in the institutions where the research was being conducted. Based on their opinions, when there was trust, the amount of information provided would not make a difference, and participants would join the studies.

*For example, if the researcher is working in the [referral hospital], then we should mention that, so the patients will trust [the research]. [Physician & EC member 36]*

Despite these findings, the need for participants to understand that they have a choice regarding participation in research was a key priority for stakeholders. In the survey, 82.5% (33/40) of stakeholders stated that the idea of voluntariness was essential for inclusion in the consent process, one participant and four researchers were neutral about its inclusion, and two participants thought it was not essential information. Use of the word *nghiên cứu* was considered integral to ensuring that potential study participants would realize that they could refuse to participate in the research, in contrast to medical care or a health programme, which were considered less likely to be declined in the context of this healthcare system.

*If we do not say, “It is* nghiên cứu*”, then the family may think: “What is it that you are doing?” [Not using the word research] is as if the family doesn’t have a choice, which means the doctor is forcing them to do it… I mean that people have the right to refuse, so we have to say, in the first sentence, “this is* nghiên cứu*” so that people know. [Physician & EC Member 05]*

Another parent whose child was enrolled in a dengue study agreed that keeping the word *nghiên cứu* instead of an alternative, such as programme, in the consent process was imperative, due to different connotations with the meaning of other words in the hospital setting. For these participants, programme and research have very different meanings. *Nghiên cứu* is a mechanism to “find out something” while a programme is a more standard activity within the healthcare context (e.g. Malaria Programme).

*It is better when you say* nghiên cứu *because people will understand right away that this is a topic that needs research to find out something [that we don’t know], because just saying “programme…” they won’t know what programme they’re participating in, and then they’ll say: “Oh! What programme are you inviting me into?” [Patient Representative 09]*

This is precisely why other researchers argued that the word *nghiên cứu* should be modified to increase the acceptance of joining research, as noted by a physician who had worked on clinical trials for the past 5 years.

*Ah, I think it depends on the cultural feature, the approach of the patient [the way the patient perceives it]. Because in Vietnam […] when we say* nghiên cứu *they often feel afraid…But if we just say that this is a census [questionnaires] or this is a survey, the ability of their acceptance will be higher. [Physician 27]*

The discussions circled back to the implications of the words used in the consent process (e.g. not using *nghiên cứu* to reduce fear and/or use *nghiên cứu* to help distinguish between routine care and research).

### Reimbursement, suspicions, and joining

The second theme was related to potential suspicions that could be driven by reimbursement practices and participants’ motivations for joining research. In practice, participants are reimbursed for their time and transport costs, and if enrolled in a clinical trial, Vietnamese guidelines require that the study sponsors pays all hospital bills accrued during the research. The researchers and participants had differing views about the implications of reimbursement. Researchers expressed concerns around using financial terminology in study information sheets and consent forms because in Vietnamese culture, these words sounded commercial or equivalent with being cheated.

*…we state it [payment] in so many words such as “compensation”, “cost” and “allowance”. This results in the forced and unnatural feeling for a member of the Ethical Committee and a researcher like me. [Physician & EC Member 22]*

A review of ten randomly selected consent forms used in past OUCRU studies found many different terms were used to describe various financial arrangements of the studies: reimbursement, compensation, insurance, travel costs, gift, cover, support, receive, paid, pay, cost, etc. In the hospital context, payment is normally expected for services rendered, therefore when healthcare is offered free of charge within a study, potential participants may find this practice contradictory leading to suspicions about what the research would entail, the type of treatment provided, and what would happen to collected samples.

*The reason is: when people have something for free they feel they are being cheated. It means they will wonder why it’s free? Why is it so weird? Is it dangerous or not? [Physician & EC Member 35]*

Researchers also described their views about reimbursement and its effect on motivations for participants to join research. In the researchers’ opinion, wealthier study participants were viewed as caring less about reimbursement than those with fewer resources, but the narrative was that participants cared about the money on some level, although it was not always an immediate concern.

*People usually do not care much [about reimbursement] in the period of their sickness, but after they get cured, they asked a lot. During the period of their sickness, they said, “Well, we’ve known a term like that” [it’s not important, I don’t care], but after that, people would ask in detail. Such as, how much money for clause a, clause b. [Physician 25]*

When asked directly about reimbursement, participants did not engage in the same rhetoric. Most participants stated that they did not join the study solely for the reimbursement. From survey data, potential costs of participation were considered an essential element of consent by 84.6% (11/13) of participants while only 57.1% (8/14) of participants considered information surrounding payments to be an essential item for discussion during the consent process.

Further, participants often had multiple reasons for joining the study such as altruism and/or access to (better) healthcare and diagnostics for themselves or their children.

*I may join it [research] because money is not important, they can use my child’s blood to research to make things better for society. This is important. [Patient Representative 01]*

The benefits of research participation were reported as an essential element to the consent process by 76.9% (10/13) of participants and 70.4% (19/27) of researchers. Stakeholders generally agreed that enrolling in a study provided access to benefits, beyond any reimbursement.

*I think the participants can get more benefits than those who don’t [participate]. Firstly, when the participants go to the hospital, they are examined immediately without waiting. Secondly, the doctors are specialized in dengue, so if it is true that the patients have dengue, then doctors can give a better overall conclusion of their state of illness. They are also able to anticipate the state of illness. They don’t have to wait, get tired and they also can have a full consultation. [Physician 14]*

### Whose responsibility?

When asking stakeholders about their attitudes regarding the essential components of consent processes, discussions revolved around responsibility on multiple levels: legal, parental, participant, and the research sites’ responsibilities. Researchers stressed the legal mandate to include the word *nghiên cứu* and the other ICH-GCP components as part of the consent process as a way of fulfilling both their legal research responsibility and their responsibility to participants.

*Because lawfully speaking, we have to be transparent with the patient about everything, so the issue is that we have to let the patient know that this is a research study, so if the patient accepts, then they can participate. If they don’t [accept], then they can refuse to participate. [Physician & EC Member 12]*

Stakeholders agreed that participants relied heavily on the doctors’ opinions and advice regarding medical care and involvement in research, shifting some of the participants’ decision-making to the research staff. At the complicated juncture of routine medical care and research within a large, busy hospital, the line between care and research was easily blurred. Over half (8/14) of the participants mentioned the trust they have in doctors and researchers regarding the research and their potential participation.

*We feel safe when the doctors will take care of our children’s health. We do not have medical knowledge. When the doctors guarantee 100 %, we feel safe. [Patient Representative 06]*

Another patient representative stated at the beginning of her interview that she consented for her child to join the research so she could gain knowledge about dengue prevention for her children in the future. She elaborated that she did not find certain elements of the consent process to be essential for her to know but she felt that these elements should be essential for the physicians and researchers to understand.

*I don’t care about it [information on data analysis and sharing]. However, the doctor cares about that, they research it to know much more information. In addition, they know the reasons why they research this disease. Therefore, I should know a little bit about what the disease is, why it causes an impact on people…This [information on data analysis and sharing] I think I shouldn’t know. Those are the doctor’s [responsibility]. [Patient Representative 34]*

When probed about what information she thought was necessary for her to understand, she continued:

*They give me this paper, I will read it carefully. If I have trouble with anything, I will ask them. If I feel there are benefits, I will study it deeply. Then, I will ask the doctor. [Patient Representative 34]*

Researchers suggested that potential participants often did not read all information in the consent form for a range of reasons.

*…in fact not all of the [participants] read it. […] they just say, “Can you explain it to me, and make it short and concise so I understand?” […] the educational standards of most malaria and tuberculosis patients are low and their socioeconomic conditions are too. Therefore, they just care about the immediate future, like finances, what benefits they can get, does this treatment help their child; they rarely care about the forms we ask them to sign. [Physician 10]*

When a potential participant took time to consider the implications of joining, research staff could become impatient and make assumptions about the participant’s level of understanding.

*Patients were so illiterate and they had to consider finely about their signature or consenting to take part in the research. They wanted more opinions from their two or three relatives for sure. It kept me waiting up to 3 h to get their answers. Otherwise there were some academic terms in the document they did not understand: “What is scientific research?” Then I explained about it and said that their joining was not involved with any legal responsibilities. As long as they were faced with any papers, documents or anything else, they hesitated without understanding. Ah, they would often get confused. [Physician 20]*

Research procedures and burdens (e.g. number of study visits, amount of laboratory tests required), was listed as an element that should be included in the consent form and 73.2% (30/41) of the stakeholders agreed that it was essential. Although participants relied on researchers’ advice about joining, 85.7% (12/14) of participants found their responsibilities and the research procedures essential to include in the consent process.

## Discussion

In exploring what the word research meant to participants, there was a tension between researchers’ obligations to use the term *nghiên cứu* and the desire to increase participation by changing the term altogether, due to its perceived negative connotations. While the majority of participants expressed negative views about the term *nghiên cứu* (although these did not necessarily reflect their experiences of research) there were at least two participants with more positive attitudes. There are alternative words in Vietnamese for research, although some words (e.g. programme) might change how the participant perceives study and its voluntary nature. Determining the appropriate terminology is a priority yet the phrase itself is only the starting point. Importantly, making sure participants receive enough information to make an informed choice becomes complicated when researchers limit the amount of information provided to increase participation or to make the consent process more rapid. Study participants may want more information on various aspects of the study and their lack of understanding of research may be caused by a lack of comprehensible information.

Perceptions of research reimbursements may be influenced by real and/or rumored research abuses and often are indicative of larger inequalities in the participants’ worlds and differences in their culture and value systems [17, 21]. The participants’ understandings about research and health more generally likely did not reflect an inability to understand relevant concepts, as suggested by some researchers in this study, but a gap between how researchers portray biomedical research and the participants’ varying worldviews related to health and illness [22]. Participants’ understandings of health and illness may be challenged when researchers use technical terminologies that do not map onto local realities and understanding.

Our findings suggest that the appropriate financial terminology to use in Vietnam may differ from that in other clinical research settings. The ideas surrounding reimbursement relate not only to the terms used but also to the idea that in the hospital setting, payment was usually expected, which led to a discrepancy between hospital norms and research practice. Researchers’ thought that reimbursement could lead to suspicions about the research and concerns about a commercial researcher/participant relationship. Researchers also assumed that potential participants were motivated to take part solely by the reimbursement, which was not always the case; there were usually additional motivations for participating and other perceived benefits. Similar findings regarding fair benefits were noted from a study conducted in Kenya indicating the complexities in trying to determine what is fair but not exploitative [23].

It is important to consider the broader social and economic constraints that participants face when deciding about research participation, because their responses to these constraints vary widely and could impact the voluntariness of their decisions [7]. When participants enroll in clinical studies while simultaneously holding very strong and often negative views about research, it is important to understand why and distinguish between challenges relating to understanding (e.g. therapeutic misconception) and challenges related to voluntariness (e.g. the research is the best option from a range of poor choices). For example, study participation may be seen as the best, or only, route to effective healthcare. It is important to consider the effects of external constraints on free-decision making. At what point is the study truly a social good and when do the healthcare benefits cause exploitation? [24]. Beyond the stakeholders, there is a whole realm of institutional spaces and motivations for bringing the research into the context that is frequently not considered.

The line between medical care and research is easily blurred when the research takes place within a hospital and the physicians that treat the patients are also the physicians that recruit them as research participants. In practice, it may be difficult for a patient to turn down a research opportunity from their physician, even if the physician assures them that they can choose whether or not to join the study and that their future care will not be impacted [7, 25]. Further, in the context of a hospital, participants may think that they are obtaining individualized care in a clinical trial (i.e. therapeutic misconception) when this is not the case [26]. Researchers need to help study participants distinguish between individualized care and research to promote free decision-making about participation, which could begin with sufficient information or perhaps including an expanded discussion of *nghiên cứu* instead of changing the term to reduce fear.

For many participants, trust in the healthcare providers was a significant element in decision-making about research participation. The role of trust regarding research participation has been observed in multiple settings [16, 27–29]. In other contexts, it seems that mistrust is a more important element that needs to be addressed in communities where research takes place [30, 31]. In contrast, our findings generally indicate high levels of trust, which is consistent with research conducted by Merson and colleagues [32] in Vietnam, where participants stated that they had trust in the decisions of the medical doctors regarding the future use of their data. Using trust for decision-making about research participation should be considered as valid as information based decision-making in the context of consent [33]. However, it is important that we ensure that participants choose to make decisions based on trust, rather than feeling they are unable to make decisions based more substantially on information because it is not provided in a comprehensible way or because the support from researchers is lacking. Our findings related to trust also stress the importance of having systems in place to ensure that research studies are designed and implemented ethically [34], with the understanding that these systems alone will likely not improve trustworthiness of the researchers and institutions [35]. If participants’ trust in physicians and institutions indeed plays an important role in their decision making, then we need to make sure that all stakeholders acknowledge and respond to it appropriately. The issues surrounding trust could be further explored in our context using community engaged research as a way to unpack the meaning of trust with community stakeholders and also advance public trust in research [36].

### Limitations

There were several limitations in this study including conducting the interviews and surveys at the same time, instead of at two separate time points, and conducting the full analysis after data collection was completed. It would have been useful to probe more deeply into the issues identified in the survey or in a preliminary analysis. Additionally, we interviewed the participants after they were enrolled or completed the studies, therefore we were unable to explore how their attitudes and perspectives might change during research participation. Finally, participant recruitment took place within two clinical studies related to dengue so perspectives for other types of studies and other illnesses might be varied.

## Conclusions

In this study, we examined understandings and attitudes about consent processes for research conducted in hospital environments where the lines between patient and participant and physician and researcher were quickly and easily blurred. There were many disconnects between how the researchers explained research, consent, and participation and how participants experienced it, as well as the meanings they attached to both research and the consent process. There was also a discrepancy between researchers’ knowledge about what appropriate consent meant to participants, the elements that stakeholders found essential, and views about how consent should be best sought in these contexts. These discrepancies between groups, along with the varying perspectives of all stakeholders in this study, demonstrate the importance of engagement with the hospital-based trial communities, including the potential and past participants, researchers, physicians, and hospital ethics committee members in the design, development and application of future consent processes and forms. The main findings from this study surrounding researcher-participant expectations, communication, and trust will inform future consent processes in our setting. As a starting point, we will review current guidelines and enhance researcher trainings to stress the importance of the elements of consent. We also need to acknowledge and more fully understand the important role that trust has on decision making about joining clinical studies in a hospital-based setting and its impact on the consent process.

## Supplementary information

**Additional file 1.** Question Guide.

## Data Availability

The interview transcripts are available upon request, following the standard OUCRU data sharing policy (http://www.oucru.org/data-sharing/).

## Abbreviations

CIOMS: Council for International Organizations of Medical Sciences
EC: Ethics Committee
ICH-GCP: International Conference of Harmonization Good Clinical Practice
OUCRU: Oxford University Clinical Research Unit
PI: Principle Investigator
