# Supplementary material for: Researcher and study participants’ perspectives of consent in clinical studies in four referral hospitals in Vietnam
Source: BMC Med Ethics. 2020 Jan 10;21:4. doi: 10.1186/s12910-020-0445-z (PMC6954581; doi:10.1186/s12910-020-0445-z)
Supplement: Supplementary file 1 — Additional file 1. Question Guide. [file 12910_2020_445_MOESM1_ESM.docx]

**Additional file 1**

Question Guide

| **Demographic information** | |
| --- | --- |
| 1. Full name: | 1. Gender: ⭘ Male ⭘ Female |
| 1. Interview date: [__\|__]/[__\|__]/[__\|__]   DD MM YY | |
| 1. Age: | 1. IRB member/Study doctor/Study nurse   or   1. North/south/rural/urban/grand-relative/parents |
| 1. Hospital/location: | |
| **Questions:** | |
| ***General information:***  1/ Have you ever been participating/involving in any studies?  2/ *For researcher stakeholders*: Could you briefly describe your main responsibility in that study(ies)?  *For study participant stakeholders*: Could you briefly describe what is it about?  ***Essential and important information should be included in the informed consent form for non-clinical trial studies (adapted from ICH-GCP 4.8.10)***  3/In your opinion, which piece of information as following should be necessarily included in or less necessarily excluded from the informed consent form?  [probe on all topics, as needed]  a) That the study involves research.  (b) The purpose of the study  (c) The study procedures to be followed, including all invasive procedures.  (d) The subject's responsibilities.  (e) The reasonably foreseeable risks or inconveniences to the subject and, when applicable, to an embryo, fetus, or nursing infant.  (f) The reasonably expected benefits. When there is no intended clinical benefit to the subject, the subject should be made aware of this.  (g) The alternative procedure(s) or course(s) of treatment that may be available to the subject, and their important potential benefits and risks.  (h) The compensation and/or treatment available to the subject in the event of study-related injury.  (i) The anticipated prorated payment, if any, to the subject for participating in the study.  (j) The anticipated expenses, if any, to the subject for participating in the study.  (k) That the subject's participation in the study is voluntary and that the subject may refuse to participate or withdraw from the study, at any time, without penalty or loss of benefits to which the subject is otherwise entitled.  (l) That the monitor(s), the auditor(s), the IRB/IEC, and the regulatory authority(ies) will be granted direct access to the subject's original medical records for verification of clinical study procedures and/or data, without violating the confidentiality of the subject, to the extent permitted by the applicable laws and regulations and that, by signing a written informed consent form, the subject or the subject's legally acceptable representative is authorizing such access.  (m) That records identifying the subject will be kept confidential and, to the extent permitted by the applicable laws and/or regulations, will not be made publicly available. If the results of the trial are published, the subject’s identity will remain confidential.  (n) That the subject or the subject's legally acceptable representative will be informed in a timely manner if information becomes available that may be relevant to the subject's willingness to continue participation in the studies.  (o) The person(s) to contact for further information regarding the study and the rights of study subjects, and whom to contact in the event of study-related injury.  (p) The foreseeable circumstances and/or reasons under which the subject's participation in the study may be terminated.  (q) The expected duration of the subject's participation in the study.  ***Further discussions***  4/ Which term or word in the informed consent form are considered as terminology that cause explaining difficulties for researcher and understanding difficulties for participant?  5/ [*as applicable*] For genomic studies, what is difficult to explain and how could it be improved?  6/ Do you have further contributions about the consent process and its content in general? | |
| **End interview session** | |
